# The Evolving Applications of Laparoscopic Intracorporeal Rectus Aponeuroplasty (LIRA) in Ventral Hernia Repair—A Systematic Review

**DOI:** 10.3389/jaws.2024.13497

**Published:** 2024-10-23

**Authors:** Carlos A. Balthazar da Silveira, Ana C. D. Rasador, Patrícia Marcolin, João P. G. Kasakewitch, Diego L. Lima, Salvador Morales-Conde, Flavio Malcher

**Affiliations:** ^1^ Bahiana School of Medicine and Public Health, Salvador, BA, Brazil; ^2^ Department of Surgery, Beth Israel Deaconess Medical Center, Boston, MA, United States; ^3^ Department of Surgery, Montefiore Medical Enter, Bronx, NY, United States; ^4^ Department of General and Digestive Surgery, University Hospital Virgen Macarena, Seville, Spain; ^5^ Division of General Surgery, NYU Langone Health, New York, NY, United States

**Keywords:** laparoscopic intracorporeal aponeuroplasty hernia repair ventral hernia, abdominal wall, hernia, ventral hernia, incisional hernia, laparoscopy, minimally invasive

## Abstract

**Purpose:**

Laparoscopic intracorporeal rectus aponeuroplasty (LIRA), emerged as a method that combines benefits from minimally invasive and abdominal wall reconstruction with defect closure, restoring the midline without tension by folding the posterior aponeurosis of both abdominal rectus muscles and using intraperitoneal mesh repair. We aimed to perform a systematic review of the existing evidence on LIRA results and potential applications.

**Source:**

A thorough search of Cochrane Central, Scopus, SciELO, LILACS, and PubMed/MEDLINE, focusing on studies that explored LIRA’s possible applications and results was performed. Key outcomes evaluated included recurrence, seroma, hematoma, surgical site infection (SSI), and length of hospital stay. We included both analytic data and descriptive studies.

**Principal Findings:**

Out of 128 screened studies, three met the inclusion criteria and comprised 113 patients, of which 69 (61.1%) were operated using LIRA. Three studies comprised two case series of conventional and robotic LIRA repair, and one comparative study of LIRA versus intraperitoneal underlay mesh repair (IPUM plus). No surgical site infections were reported. Seroma rates ranged between 11.1% and 50%, while no bleeding or hematoma was noted. There were no patients presenting recurrence in a median follow-up ranging from 12 to 15 months, despite the comparative study reporting a 4.4% rate of bulging without clinical recurrence. The mean length of hospital stay ranged from 12 to 36 h. LIRA presented no differences in postoperative complications compared to the IPUM plus technique.

**Conclusion:**

LIRA is linked to low recurrence and postoperative complications. It is a novel approach with potential applications in various types of primary and incisional ventral hernias.

## Introduction

Ventral and incisional hernias are among the most common conditions faced in surgical practice, with up to 2 million surgeries performed annually worldwide [1]. Within ventral hernias, midline incisional hernias (IH) are associated with increased morbidity, presenting recurrence rates of up to 50% following surgical repair [2].

Many surgical techniques have been explored to deal with ventral hernia repair (VHR), and an increase in the use of minimally invasive surgery (MIS) has been noted, especially the use of robotic platforms in the last few years [3, 4]. However, common drawbacks of MIS VHR include the difficulty of closure of the defect and pain associated with the transparietal defect closure [5, 6].

To address these limitations, the laparoscopic intracorporeal rectus aponeuroplasty (LIRA) technique emerged as a method that combines the benefits of MIS and abdominal wall reconstruction with defect closure, restoring the midline without tension nor transparietal sutures by folding the posterior aponeurosis of both abdominal rectus muscles and using intraperitoneal mesh repair [5]. In the LIRA technique, aponeurotic flaps are dissected with two incisions on the posterior fascia of both rectus muscles. The aponeurotic flaps are sutured together, with the posterior defect closure, and an intraperitoneal mesh placed to reinforce the repair. The mesh is fixed to the abdominal wall with sutures and platelet rich fibrin or tacks [5]. In this regard, we aimed to perform a systematic review of the existing evidence on LIRA results and potential applications.

## Methods

This systematic review was performed in accordance with the Preferred Reporting Items for Systematic Review and Meta-Analyses (PRISMA) Statement and recommendations from Cochrane Collaboration Handbook for Systematic Reviews of Interventions [7]. We prospectively registered our research protocol in the International Prospective Register of Systematic Reviews (PROSPERO) with the ID CRD42024559960.

### Eligibility Criteria

We included in this systematic review studies that met all the following eligibility criteria: observational or randomized controlled trial studies; with the LIRA technique being analyzed; in patients undergoing ventral hernia repair. We excluded studies with other techniques than LIRA; inguinal hernia repair; case reports, video reports, and systematic reviews; and conference abstracts. We cited studies that we found to be historically important.

### Search Strategy and Data Extraction

Two authors (C.S. and A.R.) independently and systematically searched PubMed, Embase, Cochrane Library, Scielo, and Lilacs from inception to March 10th, 2024. The following terms were used without filters, publication date, or language restrictions: (rectus aponeuroplasty OR LIRA) AND hernia. We also included all the mesh terms related to the principal search strategy terms in our search. The references from all included studies, and previous systematic reviews were also searched manually for any additional studies. Eventual conflicts were resolved by consensus among the authors. Two authors (C.S. and A.R.) independently extracted the following data from selected studies: (1) country; (2) number of patients; (3) ventral hernia type; (4) hernia width and length; (5) mesh area, and (5) follow-up. Ventral hernia type was defined according to the EHS classification [8].

### Quality Assessment

The risk of bias was assessed using the Risk of Bias in Non-randomized Studies (ROBINS-I) [9]. Two authors (A.R. and J.K.) independently assessed the risk of bias in each study and discrepancies were resolved by a third author (C.S.) after discussing the reasons for divergence.

### Endpoints

Our main outcomes were postoperative complications: Surgical site infection (SSI); seroma; and hematoma rates. Seroma was also depicted according to the Morales-Conde classification [10]. Seromas classified as equal to or higher than grade II were considered clinically relevant. We also analyzed chronic pain rates and the mean visual analog scale (VAS) at different times postoperatively. Chronic pain was defined as pain lasting for 3 months postoperatively. As additional outcomes, we analyzed the operative time, and the length of hospital stay (LOS), in days.

## Results

### Study Selection and Characteristics

**FIGURE 1:**
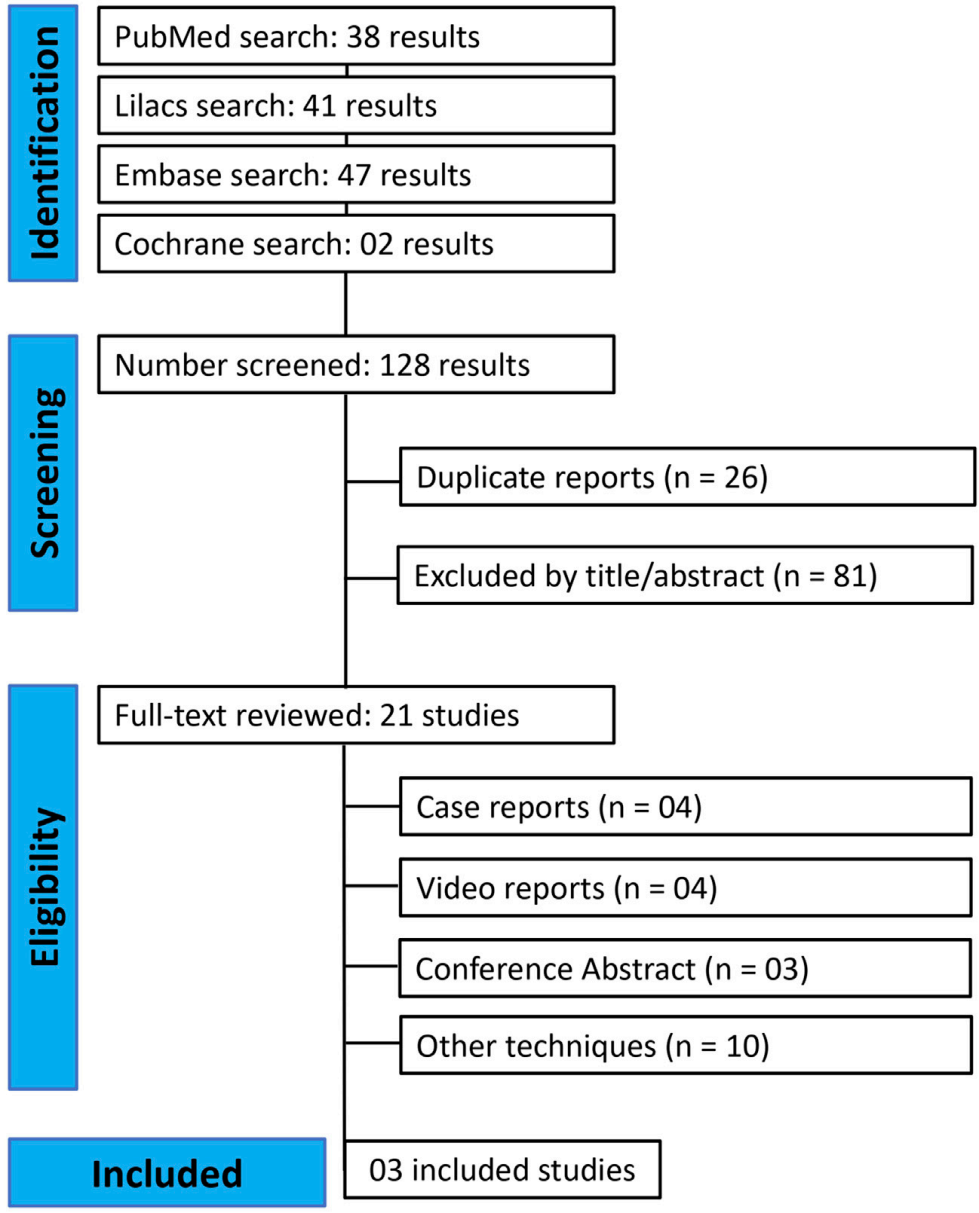
PRISMA flowchart of included studies.

The initial search yielded 128 results. After removing duplicate studies, 102 records were identified through database searching, and their summaries were screened for eligibility. Of these, 21 remained and were fully reviewed based on predefined eligibility criteria (Figure 1). A total of three studies were ultimately included, comprising 113 patients, of which 69 (61.1%) were operated using LIRA. Among the studies, two were case series of conventional and robotic LIRA repair, and one comparative study of LIRA versus intraperitoneal underlay mesh repair (IPUM plus). All the studies’ characteristics are available in Table 1.

**TABLE 1:** Individual studies’ baseline characteristics.

Study (year)	Country	Design	Sample (%)	Surgical Approach	Mean age (SD)	Mean BMI (SD)
Gómez-Menchero (2018)	Spain	Prospective cohort	LIRA: 12 (100%)	Laparoscopic LIRA	56.5 (10.5)	30.1 (5.1)
Gómez-Menchero (2024)	Spain	Retrospective cohort	LIRA: 45 (48.9%) IPUM-PLUS: 47 (51.1%)	Laparoscopic LIRA	57.5 (10.4)	32.4 (8)
Lima (2022)	USA	Retrospective cohort	LIRA: 8 (100%)	Robotic LIRA	49.25 (14.1)	31.9 (5.3)

LIRA, laparoscopic intracorporeal rectus aponeuroplasty; SD, standard deviation; BMI, body mass index.

Three studies were excluded because they were video reports analyzing LIRA for other defects than midline ventral hernias, but they were listed in the discussion. Mean hernia length ranged from 7.9 to 10.2 cm (cm). Table 1 summarizes individual studies’ characteristics. Hernia characteristics are available in Table 2.

**TABLE 2:** Individual studies’ hernia characteristics.

Study (year)	EHS hernia classification (%)	Incisional n (%)	Mean Hernia length cm (SD)	Mean hernia width cm (SD)	Mean mesh area cm^2^ (SD)
Gómez-Menchero (2018)	N/A	9 (75%)	7.9 (3)	5.5 (1.1)	N/A
Gómez-Menchero (2024)	M2: 25 (55.6%) M3: 18 (40%) M4: 2 (4.4%)	31 (68.9%)	10.2 (4.8)	6.2 (1.5)	361.8 (108.3)
Lima (2022)	M2:3 (37.5%) M3: 2 (25%) M2/M3: 2 (25%) Diastasis: 1 (12.5%)	3 (37.5%)	N/A	3.9 (1.7)	274.5 (64.2)

EHS, european hernia society; M2, epigastric; M3, umbilical; M4, infraumbilical; SD, standard deviation; Cm, centimeters.

### Quality Assessment

**FIGURE 2:**
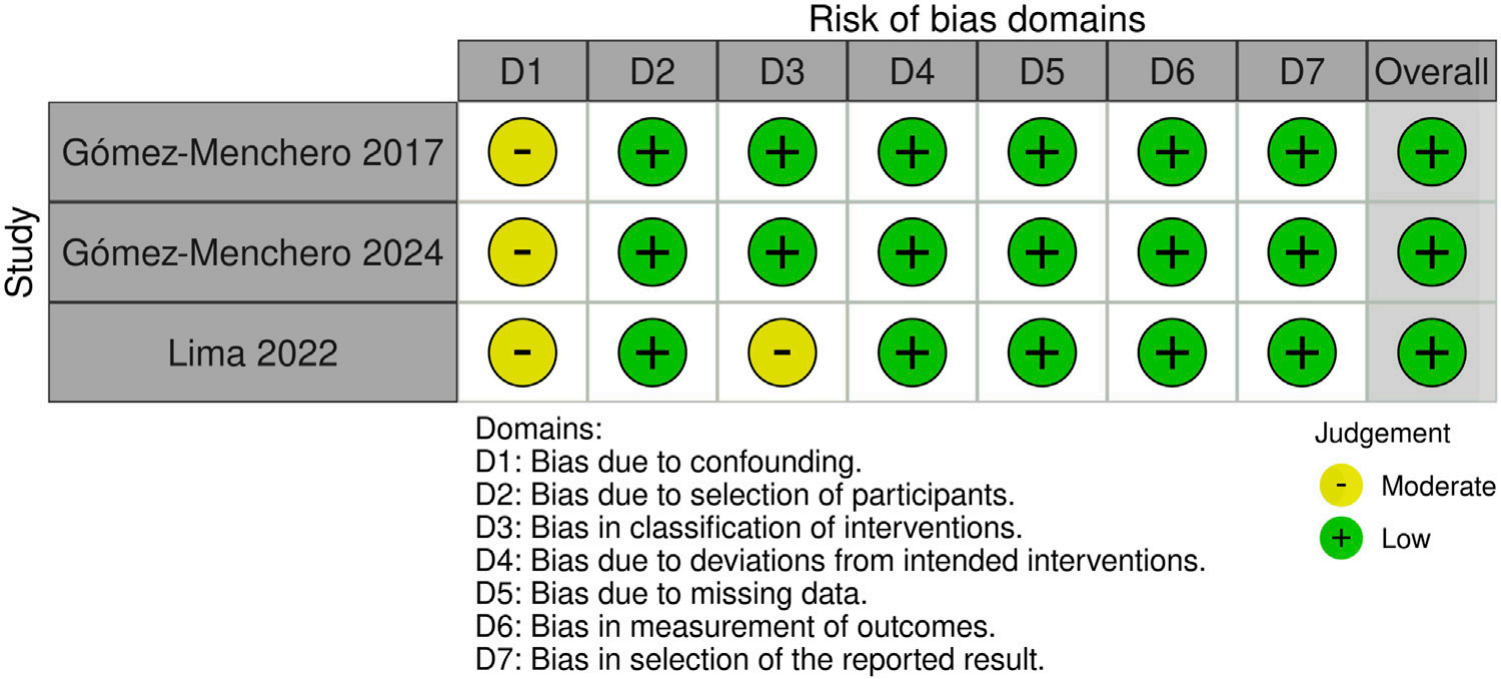
Risk of Bias assessment of included studies.

The overall risk of bias in the included studies was low. The domain in which the studies presented some concerns was the confounding factors, mostly due to the retrospective design of all the studies analyzed. The full risk of bias judgment is available in Figure 2.

### Endpoints

Postoperative complications data are available in Table 3. SSI rates were reported by two of our included studies. The overall SSI rate was 0 (0%) in both studies, comprising 20 patients analyzed. All the included studies reported data on hematoma rates, ranging from 0% to 8.3%, with the study focusing on robotic LIRA reporting no hematomas.

**TABLE 3:** Intraoperative findings and postoperative complications.

Study (year)	Mean OR time Min (SD/IQR)	Seroma n (%)	Hematoma n (%)	SSI n (%)	Mean LOS days (SD/IQR)	Recurrence n (%)	Follow-up Months (SD/IQR)
Gómez-Menchero (2018)	54 (40–75)	Total: 6 (50%) Type 0b: 2 (16.6%) Type 1: 3 (25%) Type 3a: 1 (8.3%)	1 (8.3%)	0 (0%)	1.5 (0.8–2.1)	0 (0%)	15 (12–24)
Gómez-Menchero (2024)	55.2 (9.9)	Total: 20 (44.4%) Type 0b: 4 (8.9%) Type 1: 11 (24.4%) Type 2a: 2 (4.4%) Type 3a: 3 (6.7%)	2 (4.4)	N/A	N/A	0 (0%)	12
Lima (2022)	172 (139.8-293.3)	1 (12.5%)	0 (0%)	0 (0%)	0.5 (0–1.8)	N/A	0.66 (0.53–1.53)

EHS, european hernia society; M2, epigastric; M3, umbilical; M4, infraumbilical; SD, standard deviation; Cm, centimeters.

All the studies reported data on seroma rates. Overall seroma rates, comprising all seroma grades, ranged between 12.5% and 50%. The lower rate was reported for the study analyzing robotic LIRA. However, two studies depicted seroma according to its grade, with 8.3%–11.1% rates of clinically relevant seroma.

Only Gómez-Menchero *et al* 2024 [11] presented data on chronic pain, with a rate of 4.4% for this outcome. However, two studies presented data on the VAS pain scale. Mean VAS on day 1 postoperatively ranged from 3.9 to 5 out of 10. The VAS scores ranged from 1.1 to 1.9 and 0.1 to 0.4 in 1 month and 1 week respectively. At 1 year postoperatively, both studies showed VAS scores close to 0 out of 10, ranging from 0 to 0.2. All data regarding postoperative pain are depicted in Table 4.

**TABLE 4:** Pain outcomes.

Study (year)	Chronic pain	1-Day VAS score	1-Week VAS score	1-Month VAS score	1-Year VAS score
Gómez-Menchero (2018)	N/A	3.9 (3.5)	1.1 (0)	0.1 (0)	0 (0)
Gómez-Menchero (2024)	2 (4.4%)	5 (2.1)	1.9 (1.9)	0.4 (1)	0.2 (1)
Lima (2022)	N/A	N/A	N/A	N/A	N/A

VAS, visual analog scale pain score; SD, standard deviation; N/A, data not available.

Two of the included studies analyzed recurrence rates. There were no patients presenting recurrence in a median follow-up ranging from 12 to 15 months, despite the comparative study reporting a 4.4% rate of bulging without clinical recurrence.

Mean operative time ranged from 54 to 172 min among the three studies. However, it showed interesting differences between the two laparoscopic surgeries, which ranged from 54 to 55.2 min, and robotic LIRA, with a mean operative time of 172 min. Only two studies reported the mean LOS, which ranged from 0.5 to 1.5 days for the robotic and laparoscopic LIRA respectively.

The comparative study conducted by Gómez-Menchero *et al* 2024 [11] compared LIRA technique with intraperitoneal underlay mesh repair with defect closure (IPUM plus). The only outcome that presented statistically significant differences between the groups was bulging rates, 4.4% versus 21.3% for the LIRA and IPUM plus techniques respectively (P = 0.02).

## Discussion

This systematic review with three studies and 113 patients showed that LIRA was associated with a low risk of SSI and hematoma formation, which is even more evident for the robotic approach. On the other hand, LIRA was associated with seroma rates between 12.5% and 50%. However, this rate is reduced to a maximum of 11.1% when considering clinically relevant seroma. Furthermore, it was associated with low chronic pain rates (4.4%), and a close to 0 mean VAS score at 1 year postoperatively. It was associated with a 0% recurrence in a follow-up between 12 and 15 months, with a bulging rate of 4.4%. A trend toward higher operative time and a reduced LOS was noted for the robotic approach.

One of the major concerns regarding the initially described laparoscopic technique for VHR by LeBlanc et al is the intraperitoneal mesh placement without defect closure, which can lead to an increased bulging effect after the repair [5, 12]. The first technique developed to address this issue was the repair proposed by Chalala et al., with reported bulging rates of 1.5% after defect closure via laparoscopy [13]. Following the development of this technique, other laparoscopic approaches have been created with a focus on defect closure. In 2010, Orenstein et al. described the shoelacing laparoscopic technique, which demonstrated a 0% bulging rate in a study involving 47 patients [14]. Furthermore, Clapp et al showed that closing the defect through their trans-cutaneous technique presented an 8.3% bulging rate compared to the 69.4% rate for the classically laparoscopic VHR described by LeBlanc et al (p = 0.0001) [15]. LIRA technique was associated with a 4.4% rate of bulging, representing an important improvement compared to the classic MIS repair with intraperitoneal mesh. When compared to the IPOM plus repair, Gomez-Menchero et al (2024) showed significantly lower bulging rates for LIRA repair, 4.4% versus 21.3% (p = 0.017), reinforcing the effect of LIRA technique on bulging reduction even when compared to intraperitoneal techniques with defect closure [11].

Despite addressing the issue of bulging, these techniques have been suggested as potential causes of chronic pain in larger defects, where primary defect transparietal closure may be associated with tension [16]. The study conducted by Clapp et al demonstrated no statistically significant differences in chronic pain rates when the defect is closed [15]. On the other hand, our study showed a mean VAS pain scale at 1 year postoperatively of close to 0 for LIRA technique. Also, a low chronic pain rate of 4.4% was evidenced for the LIRA technique compared to 6.4% for IPUM plus technique [11]. This finding highlights the potential of LIRA technique in reducing chronic pain rates associated with IPUM plus technique. There is also a lack of pain analysis in the study conducted by Lima et al. Considering that the other two studies may contain overlapping populations, this limits the extrapolation of pain results for the clinical practice.

Another suggested complication associated with increased tension in large defects closure is recurrence and mesh eventration [16]. A recent meta-analysis conducted by Tandon et al. demonstrated reduced rates of hernia-related events for defect closure, encompassing a combination of recurrence, mesh eventration, and bulging. However, almost all the studies included in the meta-analysis conducted by Tandon et al analyzed small defects, limiting its extrapolation to higher defects [17]. Our systematic review showed that LIRA was associated with no recurrences in a follow-up ranging between 12 and 15 months.

Despite the listed advantages of LIRA technique, a concern needs to be made regarding our findings on seroma rates, which ranged between 12.5% and 50%. Previous studies demonstrate that MIS VHR is associated with seroma rates of up to 78% [17–21]. However, current literature suggests that almost all seromas developed in the postoperative period following VHR are not of clinical relevance [22, 23]. According to Morales-Condé classification, seromas are divided based on clinical presentation, as almost all patients will develop a radiological seroma which will be reabsorbed in less than a month [10, 24]. Our systematic review found rates of 8.3%–11.1% of clinically significant seroma, being considered those that last more than 1 month postoperatively (Grade II or more). In this regard, literature on symptomatic seroma presented similar data to our findings, ranging from 8% to 12.5% [25, 26]. However, no clear association can be made between seroma formation and recurrence, and studies suggest that many seromas are reabsorbed until 3 months postoperatively [22].

Furthermore, the application of the initial LIRA technique has been extrapolated to other types of hernias. Recently, between 2022 and 2023, at least three new applications for LIRA technique have been reported [27–29]. Those reports explored LIRA’s outcomes use during parastomal, suprapubic, and lateral hernias [27–29]. Despite being case and video reports, none presented postoperative complications or recurrence during the period analyzed. Also, one of our included studies analyzed LIRA with a robotic approach [30]. Previous literature suggests that robotic surgery for VHR may be associated with reduced SSI and LOS compared to both open and laparoscopic surgery [1, 31, 32]. In our included paper, advantages were noted for reduced LOS and seroma. However, an increased operative time was evidenced, possibly highlighting its surgical technique challenges and learning curve of a new surgical technique.

It is important to highlight the inherent limitations of our study. Firstly, the literature on the LIRA technique is still very sparse, and despite conducting a comprehensive search, we found only three studies that fit our eligibility criteria. Furthermore, it is important to highlight that the pooled sample of the included studies totalized only 69 patients, limiting the power analysis and external validity. Also, the follow-up of the included studies was no longer than 15 months postoperatively, limiting the clinical relevance of recurrence findings. Also, making the sample size even smaller and increasing the risk of bias, is the possibility of overlapping between Gómez-Menchero 2017 and Gómez-Menchero 2024.

Also, among the included studies, only one compared LIRA with another technique, and no studies compared LIRA with other MIS techniques like TAPP (Transabdominal Preperitoneal), eTEP (enhanced Totally extraperitoneal), IPOM, and MILOS (Mini- or Less-open Sublay Operation). Additionally, the hernia characteristics between the studies were highly heterogeneous, encompassing primary, incisional, recurrent hernias and distasis. Also, the learning curve for this new and not widely used technique needs to be better explored. Lastly, due to the limited number of studies, a meta-analysis to demonstrate pooled results, particularly related to postoperative complications, was not feasible. However, our findings also showed a clinically important difference in important outcomes such as bulging rates, highlighting the need of more studies analyzing LIRA technique.

## Conclusion

Our systematic review of three studies and 113 patients showed that LIRA presented low SSI, hematoma and clinically relevant seroma rates, without registers of recurrence in a follow-up of up to 15 months. A trend toward low LOS and an increased operative time was evidenced for the robotic approach. This highlights the potential of LIRA to improve patient outcomes. However, it is important to highlight the low number of patients and studies published on the literature, and studies with larger samples and an increased follow-up are needed to support the applicability of LIRA for VHR.
